# Molecular Deconvolution Platform to Establish Disease Mechanisms by Surveying GPCR Signaling

**DOI:** 10.1016/j.celrep.2018.06.080

**Published:** 2018-07-17

**Authors:** Ikuo Masuho, Sreenivas Chavali, Brian S. Muntean, Nickolas K. Skamangas, Kristina Simonyan, Dipak N. Patil, Grant M. Kramer, Laurie Ozelius, M. Madan Babu, Kirill A. Martemyanov

**Affiliations:** 1 Department of Neuroscience, The Scripps Research Institute Florida, Jupiter, FL 33458, USA; 2 MRC Laboratory of Molecular Biology, Francis Crick Avenue, Cambridge CB2 0QH, UK; 3 Department of Otolaryngology, Harvard Medical School and Massachusetts Eye and Ear, Boston, MA 02114, USA; 4 Harriet L. Wilkes Honors College, Florida Atlantic University, Jupiter, FL 33458, USA; 5 Department of Neurology, Harvard Medical School and Massachusetts General Hospital, Charlestown, MA 02129, USA

## Abstract

Despite the wealth of genetic information available, mechanisms underlying pathological effects of disease-associated mutations in components of G protein-coupled receptor (GPCR) signaling cascades remain elusive. In this study, we developed a scalable approach for the functional analysis of clinical variants in GPCR pathways along with a complete analytical framework. We applied the strategy to evaluate an extensive set of dystonia-causing mutations in G protein Gαolf. Our quantitative analysis revealed diverse mechanisms by which pathogenic variants disrupt GPCR signaling, leading to a mechanism-based classification of dystonia. In light of significant clinical heterogeneity, the mechanistic analysis of individual disease-associated variants permits tailoring personalized intervention strategies, which makes it superior to the current phenotype-based approach. We propose that the platform developed in this study can be universally applied to evaluate disease mechanisms for conditions associated with genetic variation in all components of GPCR signaling.

## Highlights

• A scalable platform allows multidimensional analysis of GPCR signaling
• The approach is applied to dystonia-causing mutations in G protein Gαolf
• Pathogenic variants in Gαolf disrupt GPCR signaling by diverse mechanisms
• Mechanism-based disease classification could allow targeted therapies

## Graphical Abstract

**Figure:**
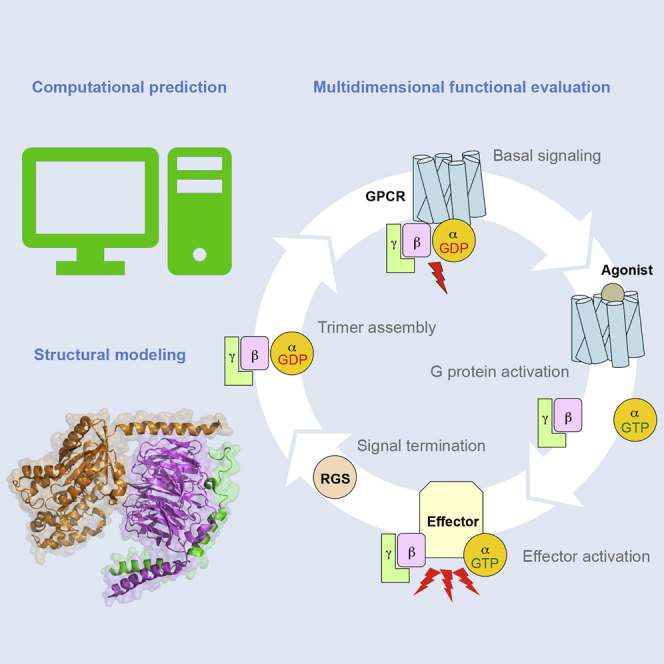

## Teaser

Masuho et al. report development of a scalable platform for functional analysis of variants in GPCR pathway components. They apply the method to interrogate molecular mechanisms of dystonia by surveying an exhaustive set of clinical variants in Gαolf revealing diversity of mechanisms underlying signaling disruptions and offering mechanism-based disease classification.

## Introduction

In recent years, breakthroughs in sequencing technology have tremendously accelerated the discovery of the genetic basis of diseases. Accordingly, genomic epidemiology is now practiced at a larger scale (Auton et al., 2015). These advances are expected to improve diagnostic results and make personalized pharmacogenomic approach an achievable goal. A key prerequisite for successfully accomplishing this is to understand how individual variants affect biological function at the molecular level.

One of the most clinically significant classes of molecules includes components of G protein-coupled receptor (GPCR) pathways, the largest gene family encoded in the human genome (Offermanns, 2003) and immensely successful drug targets (Santos et al., 2017). Accordingly, variations in GPCRs and G proteins significantly contribute to the pharmacogenomics burden, and mutations in their genes are linked to a number of diseases (Hauser et al., 2018, Insel et al., 2007, Spiegel and Weinstein, 2004). However, the complexity of molecular organization makes functional evaluation of genetic variation in GPCR signaling components in a scalable fashion not trivial.

Dystonia is a common neurological disorder that provides a pertinent model to study genetic complexity and delineate pathology of GPCR signaling. There has been tremendous progress in understanding dystonia genetics, including the recent identification of mutations in *GNAL*, a gene encoding the G protein α subunit Gαolf, as the cause of DYT25, a form of isolated dystonia (Goodchild et al., 2013). The Gαolf is selectively enriched in the striatum, where it plays key roles in mediating GPCR signaling (Hervé, 2011). Therefore, determining how mutations in Gαolf affect GPCR signaling at a mechanistic level offers an excellent opportunity to understand the molecular basis that links the disruption of neurotransmitter signaling to dystonia, paving the way to designing personalized pharmacological remedies.

In striatal neurons, Gαolf complexes with Gβ2γ7 dimer to form Golf heterotrimer that transmits signals from dopamine D1 receptor (D1R) and adenosine A2A receptor (A2AR) to type 5 adenylyl cyclase (AC5). In mice, disruption in several components of this pathway alters processing of neuromodulatory signals by striatal neurons, leading to motor deficits (Hervé, 2011). Given the central role of Gαolf in the process, it has been proposed that dystonia-related mutations in *GNAL* cause the disease by disrupting its ability to relay D1R and A2R signals. Indeed, several of the reported mutations in the Gαolf have been reported to diminish its ability to couple to D1R (Fuchs et al., 2013, Kumar et al., 2014), but the mechanisms by which they affect signaling in the context of the relevant GPCR pathway remain unknown.

In this study, we developed a comprehensive, scalable experimental platform for evaluating functional effects of clinical variants in GPCR pathways that relies on real-time optical recordings of signaling reactions, biochemical characterization, computational predictions, and structural modeling. We applied this approach to an extensive set of disease-associated variants in Gαolf, revealing distinct mechanisms by which they alter processing of D1R signaling to AC5 in the context of interactions with Gβ2γ7 dimer. On the basis of these observations, we offer a mechanism-based classification of DYT25 dystonia centered on the functional impact on cellular signaling. This allows parsing out symptomatically indistinguishable dystonia cases grouped together by phenotype-based examination only. We anticipate that the platform presented in this study will serve as a prerequisite for developing individualized therapies.

## Results

### Multi-dimensional Evaluation of Mutational Landscape in Components of GPCR Signaling Cascades

**Figure 1:**
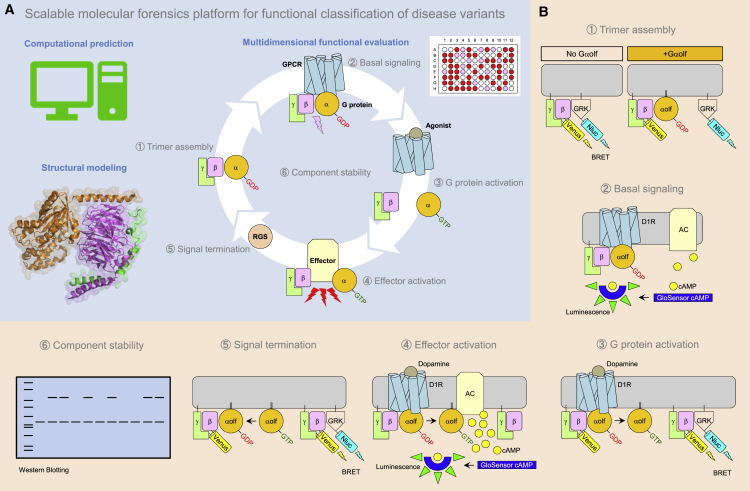
Comprehensive Analysis Platform for Pathogenic Mutations in Elements of GPCR Cascades (A) Evaluation of genetic variants were performed by computational prediction, exhaustive functional assays, and structural analysis in this study. Different steps in G protein signaling cycle investigated in this study: (1) heterotrimeric G protein assembly, (2) basal signaling, (3) agonist-induced G protein activation, (4) signaling to downstream effectors, (5) signal termination, and (6) protein stability. (B) Schematics of the assay designs.

We devised a strategy that combines computational predictions with structural analysis and an array of assays that assess various aspects of GPCR signaling (Figure 1A). In this approach, experimental evaluation is performed upon reconstitution of key pathway components into the transfected HEK293T/17 cells, where individual signaling steps of GPCR signaling are monitored by various reporter-based assays (Figure 1B). Specifically, we determined (1) the basal assembly of the heterotrimer and (2) its constitutive signaling to an effector at rest by probing protein-protein interactions with the bioluminescence resonance energy transfer (BRET) sensor and measuring basal second messenger content, respectively. (3) The ability of agonist-induced GPCR to activate G protein was studied by monitoring time course of Gα and Gβγ subunit dissociation and rearrangement by BRET, and (4) the ability of activated Gα to activate effector was assayed by monitoring kinetics of second messenger production in live cells. (5) Monitoring the kinetics of heterotrimer re-association was used to probe signal termination. (6) Finally, we independently determined the effects on protein stability by quantifying G protein expression by western blotting. All signaling assays were performed in miniaturized 96-well format using a plate reader for measuring optical readout, allowing considerable throughput and scalability. The application of this platform for the analysis of mutational landscape in G protein Gαolf is described below.

### Missense Mutations in *GNAL* Map Broadly across Multiple Structural Domains in Gαolf

**Figure 2:**
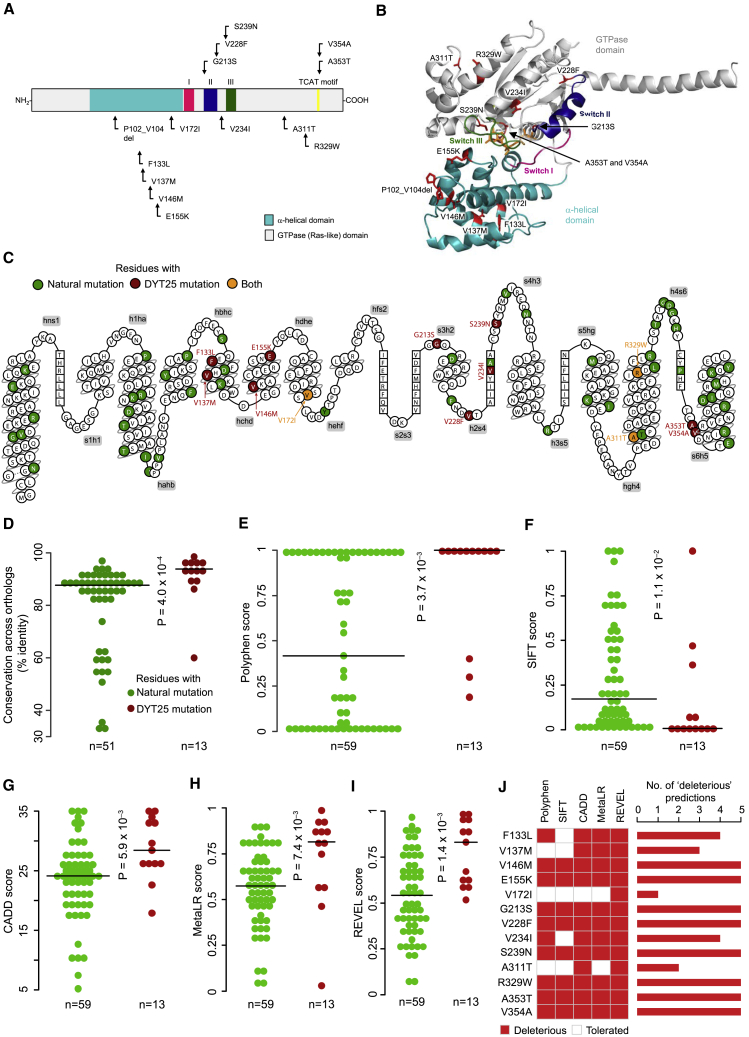
Distribution of Gαolf Mutations and Computational Analysis of Naturally Occurring Mutations in Human Gα Subunits and DYT25 Mutations (A) Mapping of the dystonia-related mutations on Gαolf sequence. (B) Structural model of Gαolf built on Gαs crystal structure (PDB: 1AZT) (Figure S2). (C) Snake plot for human Gαolf, obtained from GPCRdb (Pándy-Szekeres et al., 2018), highlighting residues with natural variants and DYT25 mutations. (D) Distribution of conservation profiles, calculated as percentage sequence identity for natural and DYT25 mutations. (E–I) Distribution of PolyPhen (E), SIFT (F), CADD (G), MetaLR (H), and REVEL (I) scores. Statistical significance was assessed using Wilcoxon rank-sum test. (J) Heatmap showing the prediction of each DYT25 amino acid substitution by different methods, with the bar plot on the right showing the number of methods predicting a given substitution to be deleterious. See also Figure S1 and Tables S1 and S2.

Although a number of mutations in *GNAL* linked to isolated torsion dystonia results in a loss of function, many variants were not evaluated for their impact on transduction of GPCR signals. Among reported mutations, splice-site alterations and frameshift and nonsense alterations in the *GNAL* coding sequence are predicted to lead to an obvious loss of function and thus were not considered in this study. The remaining 13 mutations known at the time of the study were selected for the exhaustive analysis (Table S1). In addition, we evaluated a variant, p.S239N, which was identified previously but not reported yet (Putzel et al., 2016). All of the selected mutations mapped to positions that are highly conserved among diverse vertebrate species (Flock et al., 2015) (Figure S1) and many mapped to motifs common to all Gα proteins (Figure 2A). To facilitate structural and functional studies, we generated a three-dimensional (3D) model for Gαolf on the basis of a crystal structure of a related G protein, Gαs (PDB: 1AZT) (Figure S2). We found many mutations to reside in structurally defined regions in both the α-helical and the GTPase domains (Figures 2A and 2B).

### Computational Prediction Identifies the Majority of the Isolated Dystonia Mutations to Be Deleterious

To predict the effects of DYT25 mutations, we first retrieved a list of naturally occurring missense mutations (n = 59) identified in 138,632 healthy individuals (Figure 2C) from gnomAD browser (Lek et al., 2016). We found that the amino acids mutated in DYT25 tended to be more conserved across orthologs compared with naturally occurring variants (Figure 2D). Analysis using five *in silico* tools—PolyPhen (Ng and Henikoff, 2001), SIFT (Ng and Henikoff, 2001), CADD (Kircher et al., 2014), MetaLR (Dong et al., 2015), and REVEL (Ioannidis et al., 2016)—showed that the DYT25 mutations were predicted to be deleterious more often (Figures 2E–2I; Table S2). Nevertheless, several DYT25 mutations were predicted to be tolerated, attesting to the limitations of these predictions (Figure 2J).

**Figure 3:**
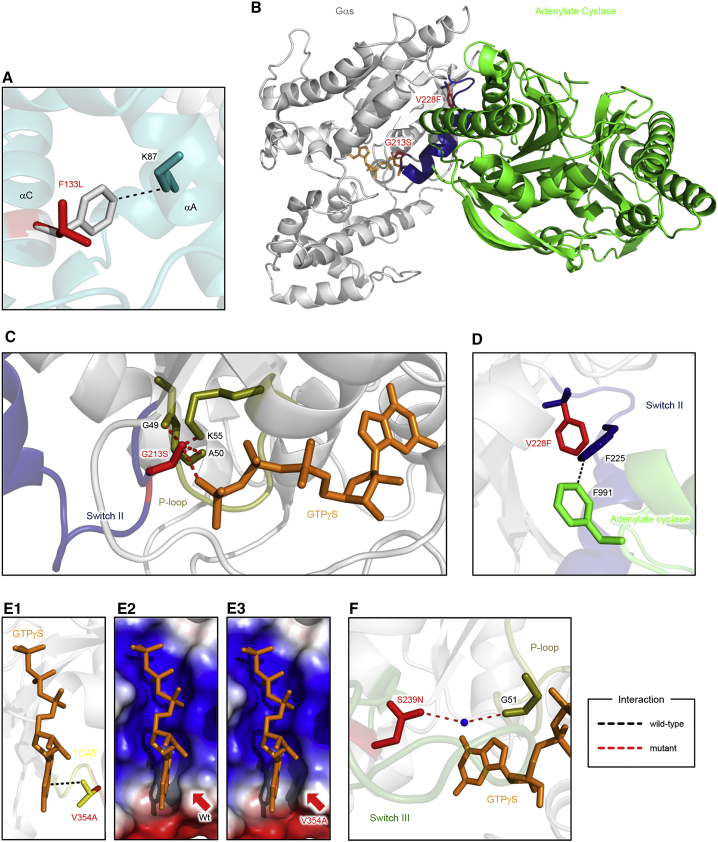
Structural Basis of Molecular Effects of F133L, G213S, V228F, V354A, and S239N Mutants (A) F133L mutation disrupts a cation-π interaction between F133 and K87. (B) Modeling the effects of G213S and V228F mutations on the crystal structure of Gαs-AC5 complex (PDB: 1AZS). (C) G213S mutation introduces hydrogen bond network with γ phosphate of GTPγS (orange) and three P loop residues (olive). (D) Predicted effects of V228F mutation on the organization of Gαs-AC5 complex. (E) V354A mutation eliminates a hydrophobic interaction with a guanine ring of a nucleotide (E1). Comparison of wild-type (E2) and V354A (E3) by electrostatic surfaces shows the broadening of nucleotide-binding pocket in the mutant. (F) S239N mutation introduces a water-mediated hydrogen bond with G51 in a P loop (olive). Please refer to detailed descriptions in the main text. See also Figure S2.

We next performed a detailed analysis by structural modeling. Several mutated amino acid residues (e.g., F133, V137, and E155) were found buried in the α-helical domain, making it likely that introducing structurally different side chains could impede Gαolf folding and/or stability. Several clusters in Gα subunits have been identified as determinants of their stability (Flock et al., 2015, Sun et al., 2015). Indeed, our structural modeling of a representative mutation in one such cluster, F133L, shows that this substitution eliminates the cation-π interaction between helices αC and αA and is thus expected to affect their packing (Figure 3A).

Alterations in several positions can also be predicted to result in functional effects. For example, G213S substitution is predicted to restrict the conformational change of Switch II, possibly compromising association with effectors (Figures 3B and 3C). Indeed, mutation of corresponding residue in Gαs (G226A) diminishes its dissociation from Gβγ and ability to stimulate AC (Lee et al., 1992). Similar effects may be induced by another mutation in Switch II, V228F, where introduction of a bulky side chain would disrupt critical contact with F991 of AC5 (Figures 3B and 3D).

Possible effects are further expected from the A353T and V354A mutations, which occur in TCAT motif (TCAV in Gαs/olf). Analogous mutations in other Gα are known to accelerate spontaneous nucleotide exchange, thus triggering GPCR-independent activation (Iiri et al., 1994, Thomas et al., 1993). Modeling suggests that the mutation V354A deprives a hydrophobic/van der Waals interaction of V354 with guanine nucleotide while broadening the size of the nucleotide-binding pocket, thus likely influencing nucleotide exchange (Figure 3E). Finally, structural modeling suggests that the S239N mutation may influence the GTPase activity by affecting the P loop conformation (Figure 3F). Overall, this bioinformatics evaluation provides valuable first-pass screening aimed at capturing salient features of variants relative to their possible structural implications, setting the stage for the experimental examination of the functional consequences.

### Mutations Compromising the Stability of Gαolf Produce Constitutively Active Gβγ Dimer

**Figure 4:**
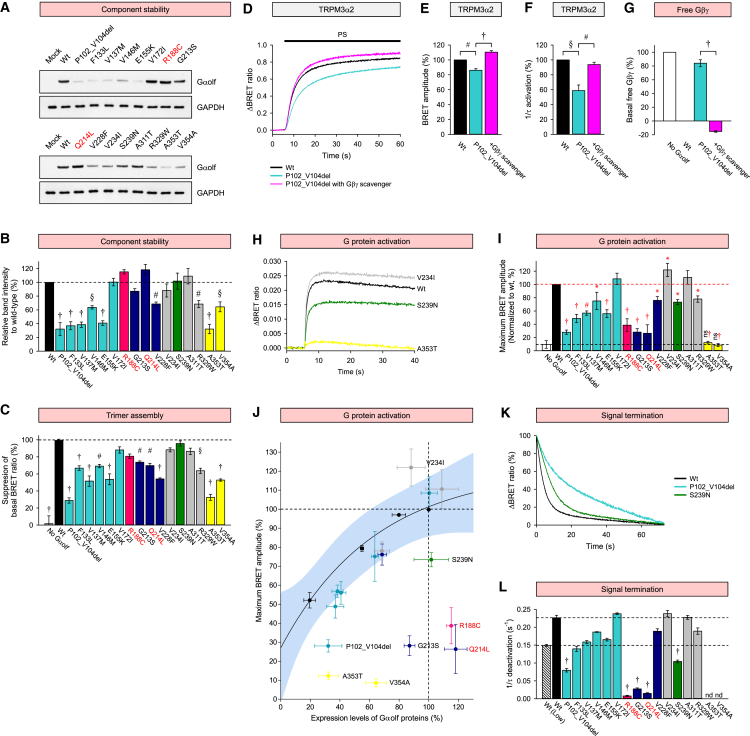
Effects of the Mutations on the Expression Levels of Gαolf Proteins, Trimer Assembly of Gαolf/Gβ2γ7, on Coupling to GPCR and Signal Termination (A) Western blotting analysis of Gαolf expression. (B) Quantification of western blotting data in (A). (C) Effect of mutations on trimer assembly measured by BRET. The ratio obtained without Gαolf is designated as 0% suppression. (D–F) Effects of P102_V104del mutation on signaling to TRPM3α2 measured by Ca^2+^ influx with the CalFluxVTN BRET-based sensor. Increased Gβγ availability inhibits Ca^2+^ influx through the TRPM3α2 channel. (D) Time course of PS-induced calcium influx through TRPM3α2. (E and F) Quantification of the response amplitude (E) and activation rates (F) relative to wild-type (WT) Gαolf. (G) Quantification of the relative amount of free Gβγ dimer. (H–J) Effect of mutations on agonist-induced G protein activation measured with masGRK3ct BRET-based sensor. (H) Time course of agonist-induced G protein activation. (I) Quantification of the maximal response amplitude. The response amplitude obtained from WT Gαolf is designated as 100%. (J) Correlation analysis of agonist-induced G protein activation versus expression level quantified from western blotting experiments. (K and L) Effect of mutations on signal termination measured as quenching BRET signal in a masGRK3ct-based system upon the addition of an antagonist. (K) Time course of signal termination upon antagonist addition. (L) Quantification of the signal termination by single exponential analysis of the time course shown in (K). Wild-type Gαolf was transfected with a low (dashed) or standard amount (black) of Gαolf to estimate fluctuation caused by variation in expression. Data are represented as mean ± SEM. See also Figure S3.

We first studied the effect of mutations on expression of Gαolf by reconstituting the D1R-Gαolf/Gβ2γ7 signaling cascade of striatal neurons in HEK293T/17 cells following optimizations for the component stoichiometry and folding (Figures S3A–S3F). We found that the expression of exogenous Gαolf mRNA and protein in transfected cells was similar to their endogenous levels in the striatum, and no endogenous Gαolf was present in untransfected HEK293T/17 cells (Figures S3G and S3H). Western blotting revealed that all mutations in the α-helical domain with the exception of V172I significantly decreased the expression of Gαolf (Figures 4A and 4B). Only four mutations in the GTPase domain (V228F, R329W, A353T, and V354A) reduced Gαolf expression, while others showed no detectable effect.

Although the loss-of-function effect is straightforward to determine, gain-of-function mutations may be harder to distinguish with this approach. Therefore, we additionally used two well-characterized constitutively active mutants (R188C and Q214L), which prevent GTP hydrolysis (Landis et al., 1989) and thus provide a useful reference for monitoring gain-of-function alterations. These mutants showed only a small increase in the expression levels (Figures 4A and 4B).

Next, we evaluated the ability of Gαolf mutants to form complexes with the Gβγ dimer. This interaction masks the effector-binding surfaces on both the Gα subunit and the Gβγ and thus prevents signal transduction (Gilman, 1987, Sprang, 2016). Therefore, loss of Gα could lead to an imbalance in the heterotrimer stoichiometry, resulting in an increased association of Gβγ with effectors. We monitored the interaction of Gβ2γ7 with its effector GRK3ct using BRET (Figure 1B). In the absence of Gαolf, most of the Gβ2γ7 is bound to GRK3ct, generating the BRET signal, which is reduced upon introduction of wild-type Gαolf because of competition for the Gβ2γ7 binding (Figure S3C). As expected, all of the mutations that decreased Gαolf expression were also less effective in suppressing GRK3ct-Gβ2γ7 interaction (Figure 4C). In addition, G213S mutant, which did not compromise Gαolf stability, showed the same effect (Figures 4B and 4C). Nevertheless, the majority of mutants exhibited normal BRET ratios, indicating uncompromised trimer formation with Gβ2γ7. Traditional biochemical pull-down assays performed with two representative mutants, P102_V104 de and A311T, confirmed validity of observations in the BRET assays (Figure S3I). These results suggest that Gαolf stability is reduced by several dystonia mutations and may yield free Gβγ dimer engaging downstream effectors at the basal state. Indeed, we observed substantial elevation of free Gβγ levels produced by a number of Gαolf mutants (Figures S3J–S3L).

To test the implications of this for the downstream signaling, we evaluated the effect of P102_104del mutant that increases basal Gβγ levels on TRPM3α2 calcium channel, a direct Gβγ effector (Dembla et al., 2017). In line with reported observations, we found that expression of Gβγ suppressed Ca^2+^ influx through TRPM3α2 (Figures S3M–S3O). The P102_104del mutant had smaller and slower Ca^2+^ influx relative to wild-type (Figures 4D–4F). Importantly, the inhibitory effect of P102_104del mutant was rescued by scavenging Gβγ, confirming that the effect is indeed mediated by increased levels of free Gβγ (Figures 4D–4G). Together, these results support a model that loss of Gαolf stability can affect signaling via increased signaling by Gβγ.

### Mutations in Nucleotide-Binding Regions Influence Coupling to GPCR and GTPase Activity

We next examined the effects of mutations on the activation and deactivation of Golf. We used an optical assay in which the stimulation of D1R induces the dissociation of Golf trimer into Gαolf and Venus-Gβ2γ7, leading to rapid increase in the BRET signal (Figure 1B). All mutants with reduced expression levels, and two mutants with normal expression (G213S and S239N) showed significantly reduced BRET amplitudes, reflecting the reduction in GPCR coupling efficiency (Figures 4H and 4I). As expected, similar deficits were also observed with the constitutively active control mutants (R188C and Q214L), validating the approach. Interestingly, two mutants with normal expression levels (V172I and A311T) showed normal amplitudes, and V234I mutant exhibited a slightly increased response.

In the case of partial effects, mutations may disproportionately affect function over the expression. To examine this possibility, we performed a correlation analysis between Gαolf expression and D1R-coupling activity by titrating wild-type Gαolf as a calibration standard and plotting agonist-induced amplitudes as a function of expression levels (Figure 4J). Using 99% confidence interval as a threshold for identifying significant deviations from the expression-activity relationship, we found that the activity of most Gαolf mutants scaled with their expression in the same manner as wild-type protein, indicating that their lower activity could be simply explained by corresponding decrease in expression (Figure 4I). However, five mutants (P102_V104del, G213S, S239N, A353T, and V354A) fell below the interval, indicating that these mutations disrupt Gαolf coupling to D1R in addition to decreasing protein stability. Interestingly, one mutant (V234I) was above the interval, suggesting an increase in the specific activity.

Because the magnitude of GPCR signaling is determined not only by the efficiency of G protein mobilization but also by the time G proteins spend in their active state, we further examined the effect of mutations on the deactivation rate of Golf following termination of D1R activity by antagonist application (Figures 1B and 4K). To ensure that fluctuations in expression of Gαolf would not affect our conclusions, we determined deactivation rate constants for wild-type Gαolf at its highest and lowest expression points, setting the threshold for significance outside of this range (Figure 4L). The control variants carrying mutations that impair GTPase activity (R188C and Q214L) dramatically reduced deactivation rates and clearly fell outside of this range. Interestingly, most of the Gαolf mutants had normal deactivation rates, with the exception of P102_V104del, G213S, and S239N (Figures 4K and 4L). In summary, we identified several pathogenic mutations in Gαolf that exhibit effects on the G protein cycle, expected to prolong the extent of the D1R signaling.

### Mutations in Gαolf Have Bidirectional Effects on Regulation of Basal and Agonist-Induced Activity of Adenylyl Cyclase

To study propagation of Gαolf signals to adenylyl cyclase (AC), we used a real-time cell-based assay measuring both baseline and agonist-induced production of cAMP (Figure 1B). Introduction of exogenous Gαolf into HEK293T/17 cells increased basal cAMP level but decreased agonist-induced cAMP production (Figure S4A), suggesting interference from the endogenous Gαs. Thus, we generated Gαs knockout cells, in which D1R activation failed to stimulate cAMP production (Figure S4B), fully rescued by introduction of Gαolf (Figures S4C and S4D).

**Figure 5:**
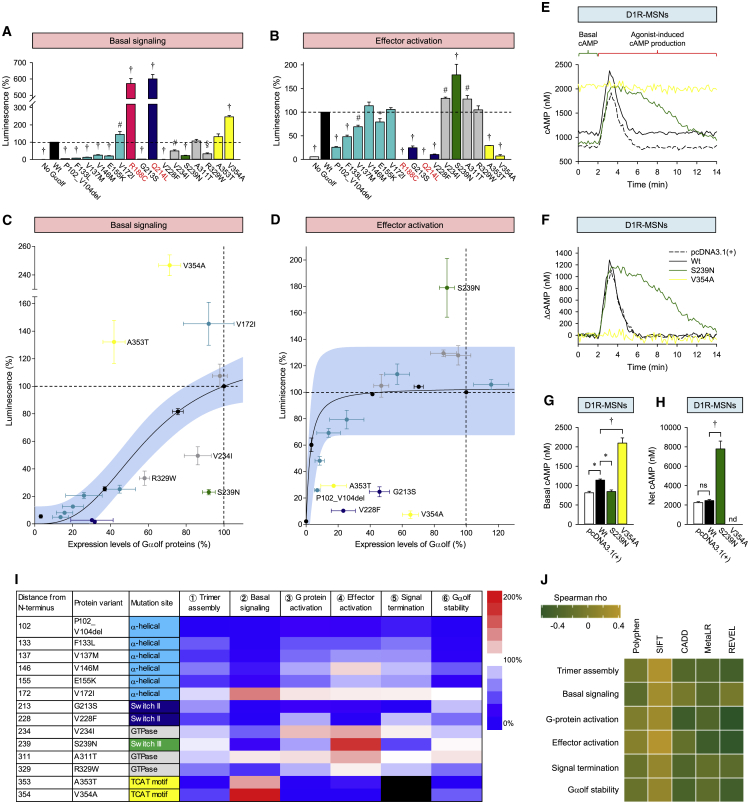
Effects of Mutations on Coupling to Adenylyl Cyclase and Functional Classification of Dystonia (A and B) Effect of mutations on basal cAMP levels (A) and agonist-induced cAMP production (B) measured with GloSensor-22F cAMP sensor. (C and D) Correlation analysis of basal cAMP (C) and agonist-induced cAMP production (D) with expression levels of Gαolf measured by quantitative western blotting. (E) Representative cAMP response of D1R-MSNs to 100 μM dopamine applied in a phasic 1 s burst at 2 min time point measured with the ^T^EPAC^VV^ sensor genetically encoded in *CAMPER* mice. (F) Normalized response from data in (E) highlighting kinetic aspects of cAMP responsiveness upon dopamine application. (G) Quantification of the basal cAMP concentration. (H) Quantification of the net cAMP change by calculating area under the curve in response to dopamine application. (I) Meta-analysis of signaling changes caused by mutations in Golf’s function combining data of all assays and measurements. (J) Heatmap showing Spearman’s rank correlation between different predictive measures of deleteriousness and experimental measures. The correlations were not statistically significant, after correcting for multiple testing using Benjamini-Hochberg method (false discovery rates). Data are represented as mean ± SEM. See also Figure S4.

Using this system, we examined the activity of Gαolf mutants on both baseline and receptor-stimulated AC activity (Figures 5A, 5B, and S4C). Most Gαolf mutants showed a clearly altered ability to regulate cAMP, in line with our observations in the BRET assays. Both constitutively active control mutants (R188C and Q214L) showed substantial elevation of the baseline and were unresponsive to agonist stimulation, as expected from saturation of the response window, validating the approach. Expression/activity correlation analysis revealed that the concentration of Gαolf scaled almost linearly with the increase in the AC activation at the baseline (Figure 5C), while the receptor-stimulated AC activity had a very narrow range of dependence on Gαolf expression (Figure 5D). At basal activity, three mutants (V234I, S239N, and R329W) exhibited diminished AC activity, whereas another three (V354A, A353T, and V172I) showed substantial enhancement (Figure 5C). When measured at the half maximal effective concentration (EC_50_) (18 ± 1.14 nM) for dopamine, at which the cAMP production did not saturate the dynamic range of the assay (Figure S4E), similar differential effects on cAMP production were also noted in the agonist-stimulated format, where S239N showed enhanced cAMP regulation, whereas five mutants were deficient (P102_V104del, G213S, V228F, A353T, and V354A) (Figure 5D). Interestingly, three mutants completely switched their behavior from inhibition to stimulation and vice versa when comparing baseline with agonist-induced mode. A353T and V354A showed a gain of function at the baseline but had clearly inhibitory effects upon D1R stimulation, whereas S239N produced low cAMP at the baseline but supported much higher dopamine-induced AC activation. The behavior of A353T and V354A were similar in direction to control R188C/Q214L mutants, suggesting an increase in constitutive activity.

Finally, we investigated two representative mutants, V354A and S239N, in the endogenous setting of D1R-expressing striatal medium spiny neurons (D1R-MSNs) using *CAMPER* reporter mice specifically expressing cAMP sensor in D1R-MSNs (Muntean et al., 2018). We observed that the V354A mutant exhibited increased basal cAMP levels compared with the wild-type; however, cAMP concentration in these neurons did not change in response to dopamine stimulation (Figures 5E–5H). In contrast, S239N exhibited lower basal cAMP compared with the wild-type while generating significantly increased cAMP production elicited by dopamine, due to prolonged cAMP response. Overall, the results in striatal neurons recapitulate functional deficiency mechanisms of the mutants observed in HEK293T/17 cells, arguing for the translatability and relevance of the observations in the model systems to elucidation of pathogenic mechanisms.

## Discussion

### Comprehensive Approach for the Analysis of Functional Consequences of Disease-Related Mutations

In this study, we examined an issue critical for understanding pathophysiology of many genetic diseases: how genetic variation alters the function of its protein product and ultimately manifests in the abnormal physiology in the disease state. It is commonly assumed that most of the mutations lead to functional deficits resulting in the loss of function. Yet it is also recognized that some mutations lead to gain of function, which likewise become pathogenic in many situations (Landis et al., 1989, Lyons et al., 1990). However, the picture is likely much more complex for the genes that encode signaling molecules, which often function by interacting with numerous partners. To further complicate matters, most of the disease genes harbor a diverse set of mutations (allelic heterogeneity), resulting in the same disorder. Therefore, understanding the exact mechanisms by which individual mutations disrupt protein function will be essential for designing effective therapeutic intervention strategies. Such an individualized strategy is already taking hold in cancer diagnostics and treatment (Daher and de Groot, 2017, Palmirotta et al., 2017), and we expect that the introduction of a robust evaluation platform for GPCR signaling may instigate the implementation of this strategy for neuropsychiatric diseases and other clinical conditions.

We took a comprehensive approach and studied virtually all reported mutations (14 in total) to date in Gαolf linked to the DYT25 form of isolated dystonia. The functional effects of mutations were evaluated in a set of cell-based assay systems that reconstituted physiologically relevant signaling partners of Gαolf. In order to perform systematic evaluation, we developed a quantitative platform for the analysis of multiple aspects of GPCR signaling, evaluating virtually an entire G protein cycle. To better understand structural and functional aspects of Gαolf biology, we further applied correlation analysis to distinguish functional effects of mutations from the effects on expression levels. Considering structural modeling data together with the comprehensive functional assessment provided insights into mechanisms of Gαolf function and underlying organization of GPCR signaling. We envision that the platform developed in this study can be used universally for deconstructing the relationship between variants in GPCR signaling cascades and human genetic diseases.

### Mutations in Gαolf Drive Dystonia via Multiple Molecular Mechanisms with Distinct Profiles of Signaling Alteration

Examining functional properties of dystonia-causing Gαolf mutants revealed surprising diversity of the underlying biochemical mechanisms. In the attempt to reconstruct the functional underpinning of the disease phenotype, we created a matrix combining all measures for each mutation in Gαolf analyzed in this study (Figure 5I). This analysis revealed that each individual mutation produced a distinct functional phenotype resulting in a distinct functional signature. In a generalized model, normally functioning GPCR pathway, at its basal state, G protein exists in a heterotrimer in which Gαolf forms a stoichiometric complex with Gβ2γ7 subunits reciprocally quenching each other’s activity. Stimulation of receptor functionally dissociates the heterotrimer, allowing both the Gαolf and Gβ2γ7 to transmit signal to their effectors: AC5 and ion channels, respectively. This signaling is balanced, and both branches are quenched simultaneously when dopamine dissociates from the receptor and the Golf heterotrimer is reassembled. We find that many mutations disrupt this balance. For example, mutations such as V234I, S239N, and A311T do not compromise heterotrimer assembly but significantly increase the overall ability of Gαolf to transmit dopamine signal. Other mutations create an imbalance in either basal activity relative to agonist-induced signaling or Gαolf to Gβγ mobilization. For example, V172I selectively enhances signaling to AC under basal conditions, whereas with V146M and R329W mutants, agonist-induced activation of AC is preserved amid deficits in Gβ2γ7 signaling both basally and in response to dopamine. On the other hand, V354A mutation produces gain in both AC and Gβγ signaling basally but diminishes it in response to dopamine.

Given the diversity of signaling disruption underlying DYT25 dystonia, we investigated whether there was an association between mutation class and clinical status and severity. For this, we compiled reported information about dystonia manifestations in all patients carrying mutations in *GNAL* that we studied (Table S1). We found that in general, there was no strong association between clinical parameters and mutation mechanisms. This is perhaps not surprising given the clinical heterogeneity, even among the members of the same family harboring the same GNAL mutation (Fuchs et al., 2013, Vemula et al., 2013). Alterations in Gαolf that result in either gain or loss of function along with mutations that produce more complex changes in signaling all produce phenotypically indistinguishable symptoms of isolated dystonia. This suggests that Golf-mediated signaling is finely tuned to a particular set point, and deviation in either direction is equally detrimental for achieving balance of neurotransmitter signaling required for movement control. We propose that regardless of their exact mechanism, Gαolf mutations are pathogenic because of the creation of signaling imbalance that deviates from the encoding logic realized by the normal protein.

The main implication of the findings presented here is that the precise mechanism of the functional effect caused by the mutations needs to be thoroughly established before attempting to correct DYT25 dystonia pharmacologically, because solutions for mitigating signaling deficits will likely be different and cannot be predicted from symptom-based clinical characterization. Such determination will have to be done experimentally, as computational algorithms are limited in their predictions (Figure 2). The amino acid sequence of Gαolf is nearly identical among vertebrate species (Figure S1), yet many functionally important sites identified in this report (e.g., F133, S239, R329, V354) are not conserved in other human Gα subunits, introducing additional challenges for *in silico* predictions. Thus, not surprisingly, we found no correlation between computational prediction and our experimental evaluation (Figure 5J). We believe that the experimental platform developed in this study should facilitate the determination of signaling mechanisms associated with pathogenic mutations in Gαolf and can be extended to assess mutations in other G protein α subunits, as well as GPCRs, Gβγ dimers, and RGS proteins, linked to diseases. Determining the fundamental function of each mutation will serve as an essential element for rational development of the corrective strategies for diseases caused by dysfunction of GPCR signaling.

## STAR★Methods

### Key Resources Table

**Table:** 

REAGENT or RESOURCE	SOURCE	IDENTIFIER
**Antibodies**		

Mouse anti-GFP (clones 7.1 and 13.1)	Roche	11814460001
Mouse monoclonal anti-GAPDH (clone 6C5)	Millipore Sigma	MAB374
Rabbit polyclonal anti-Gαolf	Corvol et al., 2001	N/A

**Chemicals, Peptides, and Recombinant Proteins**		

Dulbecco’s modified Eagle’s medium	Thermo Fisher Scientific	11965-092
Fetal bovine serum	Millipore Sigma	12303C
Sodium pyruvate	Thermo Fisher Scientific	11360-070
MEM non-essential amino acids	Thermo Fisher Scientific	11140-050
Penicillin-streptomycin	Thermo Fisher Scientific	15140-122
Puromycin	Thermo Fisher Scientific	A1113803
Neurobasal-A medium	Thermo Fisher Scientific	10888-022
GlutaMAX supplement	Thermo Fisher Scientific	35050-061
B-27 Supplement	Thermo Fisher Scientific	17504044
Hanks’ balanced salt solution	Thermo Fisher Scientific	14175-095
Papain	Worthington Biochemical	LS003126
DNase I	Thermo Fisher Scientific	18047019
Poly-D-lysine hydrobromide	Millipore Sigma	P6407
Matrigel	Corning	356230
Dulbecco’s phosphate-buffered saline	Millipore Sigma	D8537
Hanks’ balanced salt solution	Millipore Sigma	55037C
CO_2_-independent medium	Thermo Fisher Scientific	18045-088
cOmplete, EDTA-free protease inhibitor cocktail	Millipore Sigma	11873580001
Dynabeads Protein G	Thermo Fisher Scientific	10004D
Dopamine hydrochloride	Millipore Sigma	H8502

**Critical Commercial Assays**		

GloSensor cAMP Reagent	Promega	E1290
Nano-Glo Luciferase Assay Substrate (furimazine)	Promega	N1120
NucleoSpin RNA Plus Kit	Takara Bio	740984.10
Titanium One-Step RT-PCR Kit	Takara Bio	639503

**Experimental Models: Cell Lines**		

Human: HEK293T/17	ATCC	CRL11268
Human: GNAS knockout HEK293T/17	This study	N/A

**Experimental Models: Organisms/Strains**		

Mouse: CAMPER	Muntean et al., 2018	N/A
Mouse: Drd1-Cre	JAX	Tg(Drd1-cre)EY262Gsat
Mouse: C57BL/6	CRL	C57BL/6NCrl

**Oligonucleotides**		

Primer: Gαolf forward: GATCGAGAAGCAGTTGCAGAAAGAG	This study	N/A
Primer: Gαolf reverse: CTTTGTCCACTTGGAATCGTGTCTC	This study	N/A
Primer: GAPDH forward: GTCTTCACCACCATGGAGAAGG	This study	N/A
Primer: GAPDH reverse: GAAGGCCATGCCAGTGAGCTTC	This study	N/A

**Recombinant DNA**		

Plasmid: GNAS CRISPR guide RNA pLentiCRISPR v2	This paper	N/A
Plasmid: Dopamine D1 receptor	cDNA Resource Center	DRD0100000
Plasmid: Gαolf	cDNA Resource Center	GNA0L00000
Plasmid: Gαolf P102_V104del	This paper	N/A
Plasmid: Gαolf F133L	Dos Santos et al., 2016	N/A
Plasmid: Gαolf V137M	This paper	N/A
Plasmid: Gαolf V146M	This paper	N/A
Plasmid: Gαolf E155K	This paper	N/A
Plasmid: Gαolf V172I	This paper	N/A
Plasmid: Gαolf R188C	This paper	N/A
Plasmid: Gαolf G213S	This paper	N/A
Plasmid: Gαolf Q214L	cDNA Resource Center	GNA0L000C0
Plasmid: Gαolf V228F	This paper	N/A
Plasmid: Gαolf V234I	This paper	N/A
Plasmid: Gαolf S239N	This paper	N/A
Plasmid: Gαolf A311T	This paper	N/A
Plasmid: Gαolf R329W	Masuho et al., 2016	N/A
Plasmid: Gαolf A353T	This paper	N/A
Plasmid: Gαolf V354A	This paper	N/A
Plasmid: Venus 156-239-Gβ2	This paper	N/A
Plasmid: Gβ2	cDNA Resource Center	GNB0200000
Plasmid: Venus 1-155-Gγ7	Lohmann et al., 2017	N/A
Plasmid: Gγ7	cDNA Resource Center	GNG0700000
Plasmid: Flag-tagged Ric-8B	Von Dannecker et al., 2006	N/A
Plasmid: PTX-S1	Raveh et al., 2010	N/A
Plasmid: masGRK3ct-Nluc	Masuho et al., 2015b	N/A
Plasmid: CalfluxVTN	Himmelreich et al., 2017	N/A
Plasmid: pGloSensor-22F cAMP	Promega	E2301
Plasmid: mTrpM3α2-C-GFP	Ghosh et al., 2016	N/A
Plasmid: masGRK3ct	Kammermeier et al., 2000	N/A
Plasmid: pmCherry-N1 Vector	Takara Bio	632523

**Software and Algorithms**		

R version 3.4.2	R Core Team	https://www.r-project.org/
ImageJ	National Institute of Health	SCR_003070
GraphPad Prism 6	Graphpad Software	SCR_002798
SigmaPlot 12.5	SYSTAT Software	SCR_003210
PyMol	Schrödinger	SCR_000305
Clampfit 10.3	Molecular Devices	SCR_011323
T-Coffee	Notredame et al., 2000	SCR_011818
BoxShade		SCR_007165

**Other**		

GPCRdb	(Pándy-Szekeres et al., 2018	http://gpcrdb.org/
gnomAD	Lek et al., 2016	http://gnomad.broadinstitute.org/
REVEL	Ioannidis et al., 2016	https://sites.google.com/site/revelgenomics/downloads
ANNOVAR	Wang et al., 2010	http://annovar.openbioinformatics.org/en/latest/

### Contact for Reagent and Resource Sharing

Further information and requests for reagents may be directed to, and will be fulfilled by, the Lead Contact, Kirill Martemyanov (kirill@scripps.edu).

### Experimental Models and Subject Details

#### Human genetics

A patient harboring S239N mutation with isolated sporadic dystonia was screened previously (Putzel et al., 2016) but not reported as they were lost to clinical follow-up. The subject gave written informed consent, which was approved by the Institutional Review Board of the Icahn School of Medicine at Mount Sinai.

#### Cell culture

HEK293T/17 cells were chosen because of their high transfectability (Pear et al., 1993). The cells were grown in culture medium (Dulbecco’s modified Eagle’s medium supplemented with 10% fetal bovine serum, MEM non-essential amino acids, 1 mM sodium pyruvate, and antibiotics (100 units/ml penicillin and 100 μg/ml streptomycin)) at 37°C in a humidified incubator containing 5% CO_2_. This cell line is derived from a female and was purchased from ATCC.

#### GNAS knockout cell line

5 μg of GNAS CRISPR guide RNA pLentiCRISPR v2 were transfected into HEK293T/17 cells cultured on a 6-cm dish. Next day after transfection, 2.5 μg/ml puromycin was added to medium and cultured for 7 days under in the presence of puromycin. After puromycin treatment, GNAS knockout cell line was maintained in the same manner as native HEK293T/17 cells.

#### Mouse model

All experiments involving mice were approved by the Institutional Animal Care and Use Committees at the Scripps Research Institute. Experiments were conducted in accordance with the guidelines set forth by NIH. Mice (C57BL/6) were housed under standard conditions on a 12-hour light/dark cycle with continuous access to food and water in a pathogen-free facility. The CAMPER mouse was generated previously (Muntean et al., 2018). Mice were appropriately genotyped and not subject to any prior procedures. Mice used for immunoprecipitation and RT-PCR were 1 month old. Male and female mice were used across experiments.

#### Primary culture

Primary striatal neurons were cultured as previously described (Muntean et al., 2018). Brains from D1RCre-CAMPER pups (postnatal day 0) were rapidly excised followed by dissection of striata in ice cold Hanks’ Balanced Salt Solution (HBSS) (Thermo Fisher Scientific) supplemented with 20% FBS, 4.2 mM NaHCO_3_, and 1 mM HEPES. After washing tissue three times in HBSS absent of FBS, digestion was performed for 20 minutes at 37°C in a solution consisting of 137 mM NaCl, 5 mM KCl, 7 mM Na_2_HPO_4_, 25 mM HEPES, and 0.3 mg/ml Papain at pH 7.2. The tissue was next washed three times in HBSS (20% FBS), three times in HBSS (no FBS), and three times in growth media (Neurobasal-A supplemented with 2 mM GlutaMAX, 2% B27-supplement, and 1% PenStrep). Tissue was subsequently triturated by pipette in growth media in the presence of 0.05 U/μl DNase I followed by filtration through a 40 μm cell strainer and plated on poly-D-lysine (mol wt 70,000-150,000) coated glass coverslips. Neuronal cultures were maintained at 37°C in a 5% CO_2_ humidified incubator. Every three days half of the growth media was replenished with growth media absent PenStrep. Cultures were transfected one day prior to imaging experiments using Lipofectamine 2000.

### Method Details

#### Genetic constructs

Dopamine D1 receptor (GenBank NM_000794 with one silent SNP (A1263G)), Gαolf (GenBank AF493893), Gβ2 (GenBank NM_005273) and Gγ7 (GenBank AF493874) in pcDNA3.1(+) were purchased from cDNA Resource Center. Gαolf mutants were generated by site directed mutagenesis. Amino acids 156-239 of Venus was fused to a GGSGGG linker at the N terminus of Gβ2 (GenGank AF501883) to construct Venus 156-239-Gβ2. Flag-tagged Ric-8B (GenBank NM_183172 with one missense mutation (A1586G)) in pcDNA3.1 was a gift from Dr. Bettina Malnic (Von Dannecker et al., 2006). masGRK3ct-Nluc, Venus 1-155-Gγ7 (GenBank AF493874), PTX-S1, and CalfluxVTN constructs were reported previously (Himmelreich et al., 2017, Lohmann et al., 2017, Masuho et al., 2015b, Raveh et al., 2010). The guide RNA sequence (CCCCGAGAACCAGTTCAGAG) targeting human *GNAS* was cloned into an pLentiCRISPR (v2) by GenScript. pGloSensor-22F cAMP plasmids (GenBank GU174434) was purchased from Promega (Binkowski et al., 2011). pCAGGSM2-mTrpM3α2-C-GFP was a gift from Dr. Thomas Voets (Ghosh et al., 2016). The construct of masGRK3ct in a mammalian expression vector was a kind gift from Dr. Nevin A. Lambert (Kammermeier et al., 2000).

#### Antibodies

GFP (clones 7.1 and 13.1) and GAPDH (clone 6C5) antibodies were purchased from Roche and Millipore, respectively. Anti-Gαolf antibody was reported previously (Corvol et al., 2001).

#### Transfection

6-cm culture dishes were coated during incubation for 10 min at 37°C with 2.5 mL of Matrigel solution (approximately 10 μg/ml growth factor-reduced Matrigel in culture medium). Cells were seeded into the 6-cm dishes containing Matrigel solution at a density of 4 × 10^6^ cells/dish. After 4 hr, expression constructs (total 10 μg/dish) were transfected into the cells using PLUS (10 μl/dish) and Lipofectamine LTX (12 μl/dish) reagents. For BRET assay, dopamine D1 receptor, Gαolf, Venus-156-239-Gβ2, Venus-1-155-Gγ7, Flag-Ric-8B, masGRK3ct-Nluc, and PTX-S1 constructs were used at a 1:6:1:1:1:1:1 ratio (ratio 1 = 0.42 μg of plasmid DNA). For CalfluxVTN Ca^2+^ assay, mTrpM3α2-C-GFP, Gαolf, Gβ2, Gγ7, Flag-Ric-8B, PTX-S1, masGRK3, and CalfluxVTN were used at a 1:6:1:1:1:1:12:1 ratio (ratio 1 = 0.42 μg of plasmid DNA). For cAMP assay, dopamine D1 receptor, Gαolf, pGloSensor-22F cAMP, Flag-Ric-8B, and PTX-S1 constructs were used at a 1:6:6:1:1 ratio (ratio 1 = 0.42 μg of plasmid DNA). Since promiscuous nature of G protein-coupling of GPCRs are reported (Himmelreich et al., 2017, Masuho et al., 2015b), construct carrying catalytic subunit of pertussis toxin PTX-S1 were transfected to inhibit the possible coupling of endogenous Gi/o to D1R. This ensures that all signal recorded in these assays is generated exclusively by activation of Golf. According to the previous observation that Gαolf required co-expression with molecular chaperones for the formation of functional G protein complexes, Gαolf was co-transfected with Ric-8B (Chan et al., 2013, Gabay et al., 2011, Masuho et al., 2015b). Empty vector pcDNA3.1(+) was used to normalize the amount of transfected DNA.

#### BRET experiments

Agonist-dependent cellular measurements of BRET between Venus-Gβ2γ7 and masGRK3ct-Nluc were performed to examine activation of G proteins signaling in living cells (described in detail in (Masuho et al., 2015a, Masuho et al., 2015b)). Sixteen to twenty-four hr post-transfection, HEK293T/17 cells were washed once with BRET buffer (Dulbecco’s Phosphate-Buffered Saline (PBS) containing 0.5 mM MgCl_2_ and 0.1% glucose) and detached by gentle pipetting over the monolayer. Cells were harvested with centrifugation at 500 g for 5 min and resuspended in BRET buffer. Approximately 50,000 to 100,000 cells per well were distributed in 96-well flat-bottomed white microplates (Greiner Bio-One). The Nluc substrate, furimazine, were purchased from Promega and used according to the manufacturer’s instruction. BRET measurements were made using a micro plate reader (POLARstar Omega; BMG Labtech) equipped with two emission photomultiplier tubes, allowing us to detect two emissions simultaneously with highest possible resolution of 20 ms per data point. All measurements were performed at room temperature. The BRET signal is determined by calculating the ration of the light emitted by the Venus-Gβ2γ7 (535 nm with a 30 nm band path width) over the light emitted by the masGRK3ct-Nluc (475 nm with a 30 nm band path width). The average baseline value (basal BRET ratio) recorded prior to agonist stimulation was subtracted from the experimental BRET signal values to obtain ΔBRET ratio. The largest ΔBRET ratio was plotted as maximum BRET amplitude.

#### CalfluxVTN Ca^2+^ assay

Sixteen hr post-transfection, transfected cells were washed once with PBS containing 5 mM EDTA and treated with 0.025% trypsin until cells were detached from dishes. Approximately 50,000 to 100,000 cells per well were distributed in 96-well flat-bottomed white microplates (Greiner Bio-One) and cultured for 1 hr in CO_2_ incubator. Before BRET measurements were made, medium was replaced with HBSS (1.3 mM CaCl_2_, 0.8 mM MgSO_4_, 5.4 mM KCl, 0.4 mM KH_2_PO_4_, 4.2 mM NaHCO_3_, 137 mM NaCl, 0.3 mM Na_2_HPO_4_, and 5.5 mM glucose at pH 7.4) (Sigma). BRET experiments were performed as explained above.

#### cAMP assay

Sixteen to twenty-four hr post-transfection, transfected cells on a 6-cm dish are detached with 1 mL of CO_2_-independent medium containing 10% FBS. 25 μL of the cell suspension was transfered to each well of 96-well plates containing 25 μL of 2X GloSensor cAMP Reagent. 2X GloSensor cAMP Reagent was prepared according to the manufacturer’s instruction. Following incubation for 2 hr at room temperature, luminescence was monitored continuously on a POLARstar Omega at room temperature. 50 μL of dopamine was applied to cells.

#### Immunoprecipitation assay

HEK293T/17 cells in 6-cm dishes were transfected with the indicated constructs. Sixteen hr after transfection, cells were washed once with PBS and lysed with 0.5 mL of ice-cold lysis buffer (50 mM Tris, pH 7.4, 1% Triton X-100, 300 mM NaCl, 10 μM GDP, 5 mM MgCl_2_ and cOmplete, EDTA-free protease inhibitor cocktail) by sonication on ice. The resultant whole cell lysates were incubated for 30 min at 4°C with rotary agitation to solubilize membrane proteins. After lysis, cell lysates were centrifuged at 14,000 g for 15 min at 4°C. A 5 μg/sample of anti-GFP antibody and 20 μL of Dynabeads Protein G were added, and the supernatants were tumbled for 1 h at 4°C. After three washes with 1 mL of ice-cold wash buffer (50 mM Tris, pH 7.4, 1% Triton X-100, 300 mM NaCl, 10 μM GDP, 5 mM MgCl_2_ and 0.5mM phenylmethanesulfonyl fluoride), proteins bound to the beads were eluted with SDS-sample buffer (50 mM Tris, pH 6.8, 1% SDS, 143 mM β-mercaptoethanol, 0.08 mg/ml bromphenol blue, 10% glycerol). Immunoprecipitates were subjected to SDS-PAGE, transferred to polyvinylidene difluoride (PVDF) membranes, and probed with the indicated antibodies.

#### Western blotting

For each sample, about 5 × 10^6^ cells were lysed in 500 μl of sample buffer (125 mM tris-HCl, pH 6.8, 4 M urea, 4% SDS, 10% 2-mercaptoethanol, 20% glycerol, bromophenol blue (0.16 mg/ml)). Western blotting analysis of proteins was performed after samples were resolved by SDS–polyacrylamide gel electrophoresis and transferred onto PVDF membranes. Blots were blocked with 5% skim milk in PBS containing 0.1% Tween 20 (PBST) for 30min at room temperature, which was followed by a 90-min incubation with specific antibodies diluted in PBST containing 1% skim milk. Blots were washed in PBST and incubated for 45 min with a 1:10,000 dilution of secondary antibodies conjugated with horseradish peroxidase in PBST containing 1% skim milk. Proteins were visualized on X-ray films by SuperSignal West Femto substrate (Pierce). Western blotting was performed with BlotCycler automated western blot processor (Precision Biosystems, Mansfield, MA).

#### Isolation of total RNA and RT-PCR

Total RNA was purified from HEK293T/17 cells and adult mouse striatum using NucleoSpin RNA Plus Kit. Reverse transcription-PCR (RT-PCR) was performed using Titanium One-Step RT-PCR Kit using 100 ng of total RNA in 50 μL reactions for 40 cycles. Reverse transcription was performed at 50°C for 1 hr. PCR cycle conditions were 1 cycle at 94°C for 5 min; 40 cycles of 94°C for 30 s, 65°C for 30 s, 68°C for 1 min; and then 1 cycle at 68°C for 1 min. PCR primers were designed to detect Gαolf and GAPDH in both human cell line and mouse tissue. The primers were: Gαolf forward primer 5′-GATCGAGAAGCAGTTGCAGAAAGAG-3′, Gαolf reverse primer 5′-CTTTGTCCACTTGGAATCGTGTCTC-3′, GAPDH forward primer 5′-GTCTTCACCACCATGGAGAAGG-3′, GAPDH reverse primer 5′-GAAGGCCATGCCAGTGAGCTTC-3′.

#### FRET imaging

Realtime intracellular cAMP concentrations were recorded from DIV14-18 cultured striatal neurons by transferring coverslips to a microscope chamber and perfusing at 2 ml/min with a room temperature recording buffer at pH 7.3 consisting of: 1.3 mM CaCl_2_, 0.5 mM MgCl_2_, 0.4 mM MgSO_4_, 5.3 mM KCl, 0.4 mM KH_2_PO_4_, 4.2 mM NaHCO_3_, 138 mM NaCl, 0.3 mM Na_2_HPO_4_, 5.6 mM Glucose, and 20 mM HEPES. Images were acquired every 10 s on a Leica TCS SP8 MP confocal microscope through a 25X water immersion objective lens. Excitation of mTurquoise was achieved with a 442 nm diode laser paired with simultaneous 465-505 nm (mTurquoise) and 525-605 nm (Venus) bandpass emission filtration. Multiple Z stacks were captured at each time point and FRET ratios from neuronal cell bodies were calculated using ImageJ and converted to cAMP units from a calibration curve as previously described (Muntean et al., 2018). Dopamine was administered at 100 μM in phasic 1 s pulses through an SF-77B perfusion apparatus (Warner Instruments, Hamden, CT).

#### Bioinformatics

The sequence alignments were generated with T-Coffee (http://tcoffee.crg.cat/apps/tcoffee/do:regular) (Notredame et al., 2000) and colored by BoxShade (http://www.ch.embnet.org/software/BOX_form.html). Rare natural missense mutations (Minor Allele Frequency, MAF < 0.1%) in 138,632 unrelated individuals were obtained from gnomAD browser (Lek et al., 2016). Percentage identity, depicting conservation at each mutated residue position, was estimated by generating a multiple sequence alignment with MAFFT (Katoh and Standley, 2013) using one-to-one orthologs of human Gαolf protein from 64 species ranging from mammals to fungi obtained from OMA browser (Altenhoff et al., 2018). Deleteriousness scores of natural and DYT25 amino acid substitutions were predicted using PolyPhen (Adzhubei et al., 2010), SIFT (Ng and Henikoff, 2001), CADD (Kircher et al., 2014), MetaLR (Dong et al., 2015) (obtained using ANNOVAR (Wang et al., 2010) and REVEL (Ioannidis et al., 2016). The criteria used to classify a variation as deleterious or tolerated is provided in Table S2.

### Quantification and Statistical Analysis

The rate constants (1/τ) of the activation and deactivation phases were obtained by fitting a single exponential curve to the traces using Clampfit Ver. 10.3. A one-way ANOVA followed by the Fisher’s LSD post hoc test was conducted to determine the effect of mutations on the function of Gαolf subunit with GraphPad Prism Ver. 6. Asterisks indicate a significant effect of mutations compared with wild-type Gαolf (^∗^, p < 0.05; #, p < 0.01; §, p < 0.001; †, p < 0.0001). Values represent means ± SEM from three independent experiments each performed with four or six replicates.

## Supplemental Information

Document S1. Figures S1–S4 and Tables S1 and S2

Document S2. Article plus Supplemental Information
