# Supplementary material for: Molecular Deconvolution Platform to Establish Disease Mechanisms by Surveying GPCR Signaling
Source: Cell Rep. 2018 Jul 17;24(3):557–568.e5. doi: 10.1016/j.celrep.2018.06.080 (PMC6077248; doi:10.1016/j.celrep.2018.06.080)
Supplement: Document S1. Figures S1–S4 and Tables S1 and S2 [file mmc1.pdf]

Cell Reports, Volume *24*

Supplemental Information

Molecular Deconvolution Platform to Establish Disease Mechanisms by Surveying GPCR Signaling

Ikuo Masuho, Sreenivas Chavali, Brian S. Muntean, Nickolas K. Skamangas, Kristina Simonyan, Dipak N. Patil, Grant M. Kramer, Laurie Ozelius, M. Madan Babu, and Kirill A. Martemyanov

SUPPLEMENTAL FIGURES

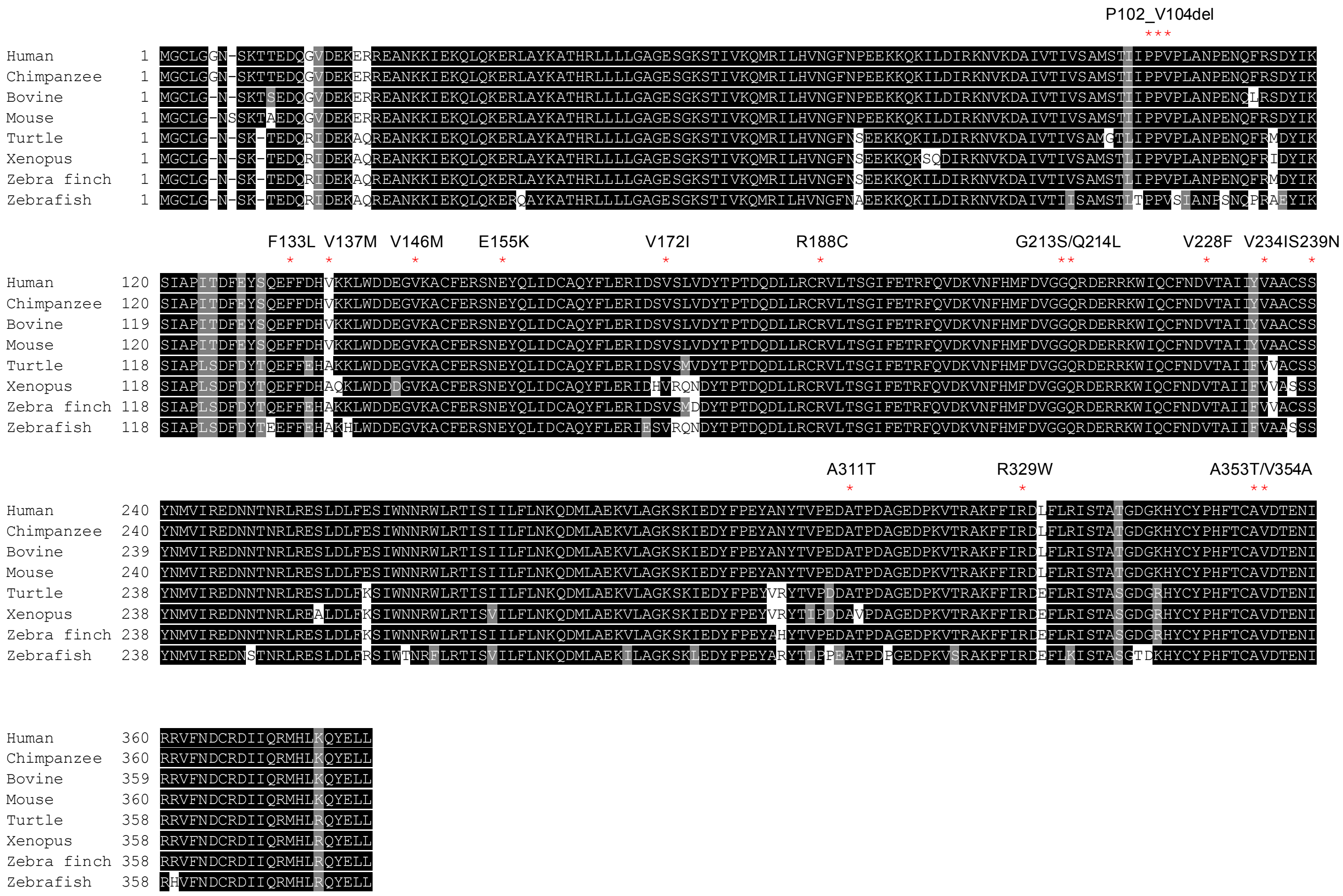

Figure S1. Homology of Gαolf subunits across different species. Related to Figure 2.

Multiple protein sequence alignment of Gαolf subunits across different species was performed. The positions of mutations are indicated by red asterisks and the corresponding amino acid changes are shown on top of the asterisks. GenBank accession numbers are AF493893 (human), XM_009433628 (chimpanzee), NM_001102554 (bovine), NM_010307 (mouse), XM_005282662 (turtle), NM_001085849 (xenopus), XM_002189478 (zebra finch), and NM_001007339 (zebrafish).

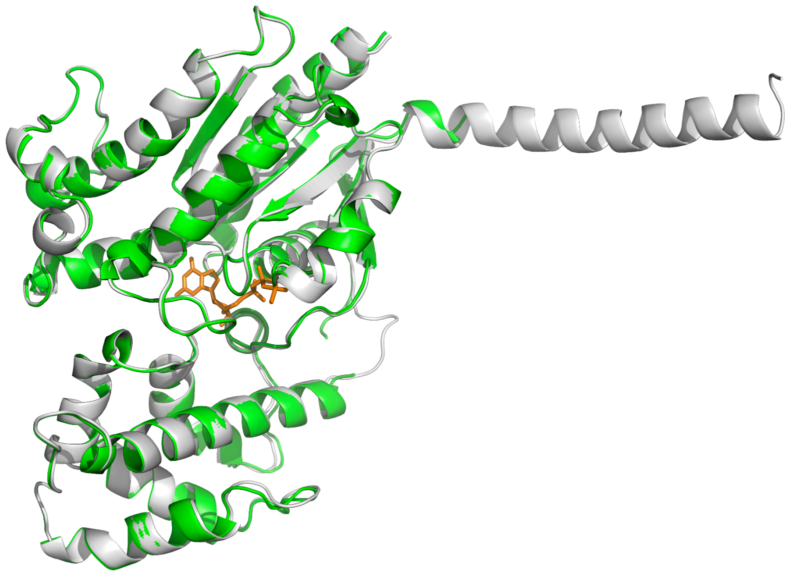

Figure S2. Evaluation of the structural model of Gαolf. Related to Figure 3.
Since Gαolf shares 88% amino-acid identity with Gαs, the homology model of Gαolf was constructed on the basis of the crystal structure of the Gαs (1AZT). The Gαs crystal structure (green) and the Gαolf model (gray) were overlaid. The Gαolf model results in root mean square deviations of 0.31 Å, indicating a good fit between the model and the reported structure.

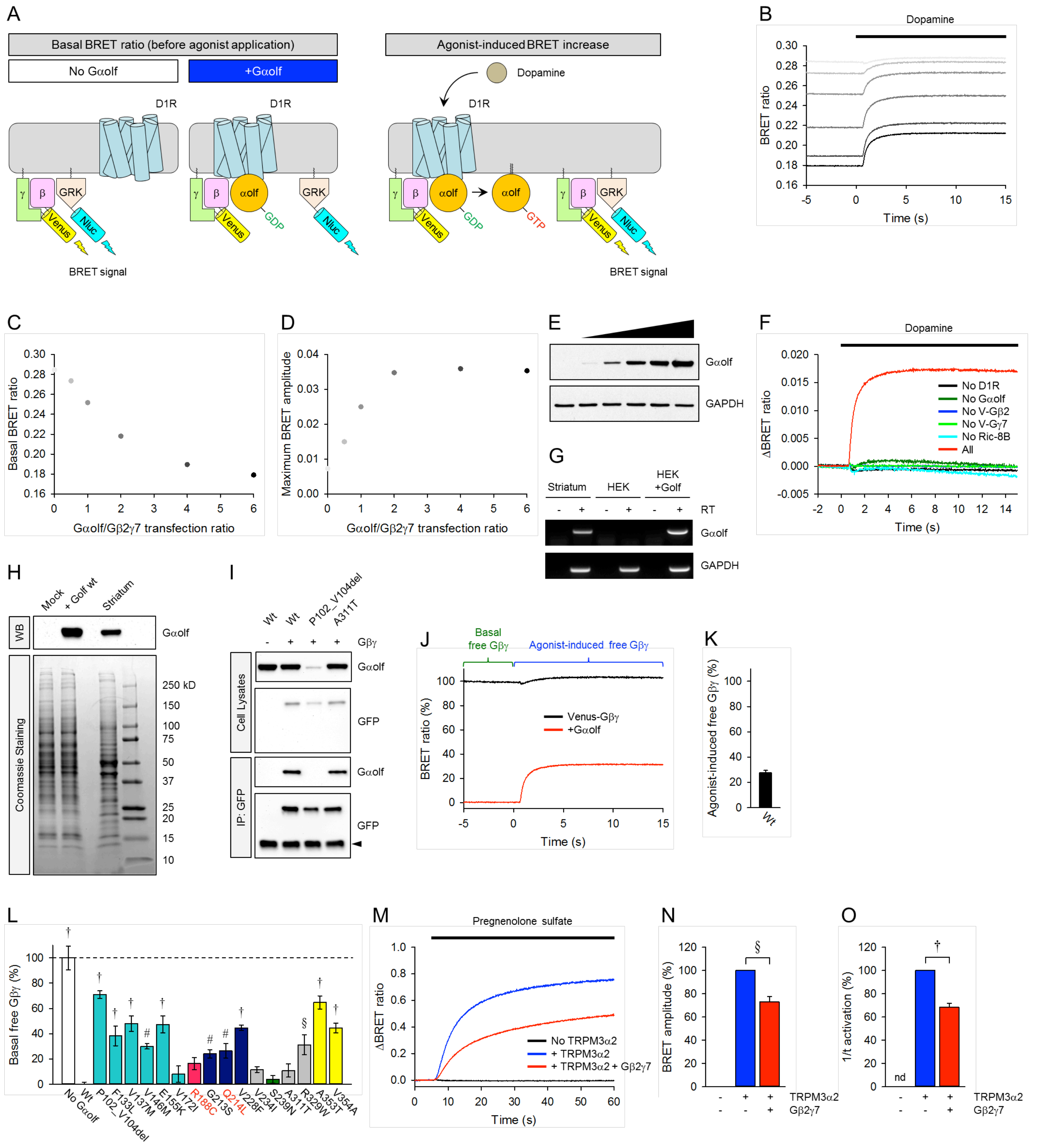

Figure S3. Reconstitution of D1R-Gαolf/Gβ2γ7 pathway in HEK293T/17 cells and effects of Gαolf mutations on Gβγ signaling. Related to Figure 4.

A, The assay design for optimizing stoichiometric Gαolf/Gβ2γ7 trimer. In the absence of exogenous Gα subunit, expression of masGRK3ct-Nluc with Venus-Gβ2γ7 produces masGRK3ct-Nluc-bound Venus-Gβ2γ7, and

results in high basal BRET signal. In this condition, agonist application does not increase BRET signal because there is no functional trimer formation. Additional expression of Gαolf sequesters Venus-Gβ2γ7 from masGRK3ct-Nluc and decreases the BRET signal. Under optimal condition, agonist application induces robust BRET increase, indicating Gαolf/Gβ2γ7 trimer formation. Therefore, the transfection condition producing 1:1 ratio of Gαolf and Gβ2γ7 is expected to minimize basal BRET ratio (before agonist application) and maximize agonist-induced BRET response. *B-E*. Experimental optimization of the stoichiometry of Gαolf and Venus-Gβ2γ7. HEK293T/17 cells were transfected with plasmids encoding D1R, Gαolf, Venus-Gβ2γ7, masGRK3ct-Nluc. ***B***, The stoichiometry of Gαolf and Venus-Gβ2γ7 were optimized by titrating the amount of Gα subunits against a constant amount of Venus- Gβ2γ7. Effect of increasing Gα with constant Venus-Gβγ for transient transfection on the basal BRET ratio and the agonist-induced maximum BRET amplitude were examined. 100 μM dopamine was applied to the transfected cells. ***C-D***, Basal BRET ratios and maximum BRET amplitudes are plotted as a function of the ratio of the amount of the Gα subunit construct to the amount of the Venus-Gβ2γ7 construct used for transfections, in the absence (basal BRET) (***C***) and presence (maximum amplitude) of a saturating concentration of dopamine (100 μM) (***D***). Each data point represents the mean ± SEM of twelve replicates. Graphs shown here are the representative data from two independent experiments with similar result. ***E***, Western blot analysis was performed with anti-Gαolf antibody. GAPDH was also probed with a specific antibody as a loading control. ***F***, Verification of the reconstitution of D1R-Gαolf/Gβ2γ7 signaling. Each of the signaling molecule was removed from the optimized transfection condition and the BRET assay was performed with transfected cells. ***G***, Semiquantitative analysis of Gαolf mRNA in the striatum (*left*) and HEK293T/17 cells without (*middle*) or with transfection of Golf (*right*) were performed by RT-PCR. Reverse transcriptase was heat-inactivated under the minus (-) condition. The heat inactivation step was omitted under the plus (+) condition. RT-PCR with specific primers for GAPDH was performed as a control. ***H***, Expression levels of Gαolf protein in HEK293T/17 cells without (*left*) or with transfection of Golf (*middle*), and the striatum (*right*) were determined by Wester blotting (*bottom*). Coomassie brilliant blue staining of a SDS-PAGE gel loaded with 10 μg of cell or tissue lysate were performed as a loading control (*top*). ***I***, Coimmunoprecipitation was performed with cells transfected with Gαolf with or without Venus-Gβγ. Transfection conditions were indicated above. The bands indicated by an arrow head are antibody light chains, showing the same amount of anti-GFP antibody was used for immunoprecipitation. ***J***, Percentage of agonist-induce free Gβγ dimer. Basal BRET ratio under the presence of stoichiometric trimer formation was presented as 0% free Gβγ. Maximum free Gβγ dimer was determined by transfection without Gαolf. Percentage of agonist-induced free Gβγ dimer was plotted as a bar graph (***K***). ***L***, Percentage of free Gβγ dimer produced by mutant Gαolf subunits before agonist application was determined and plotted as a bar graph (n = 3 independent experiments).

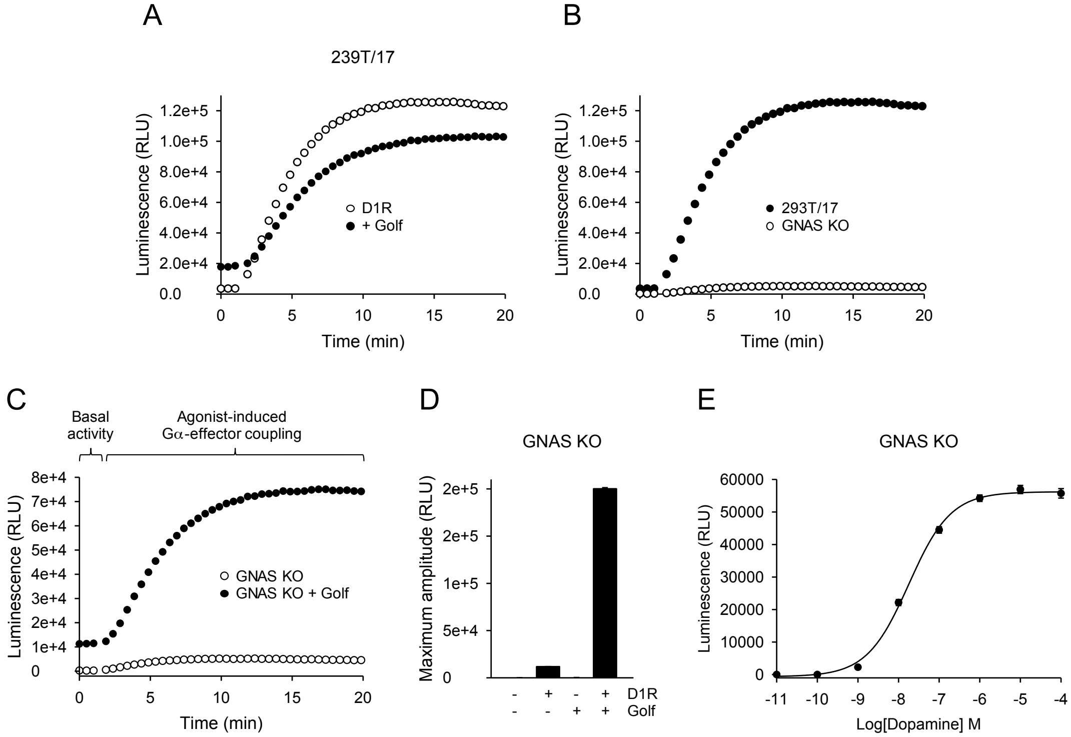

Figure S4. Generation of a GNAS knockout cell line and reconstitution of D1R-Golf-AC signaling in the cells. Related to Figure 5.

A, Agonist-induced cAMP production detected with GloSensor cAMP sensor. The agonist-induced cAMP production of HEK293T/17 cells transfected with (filled circle) or without (open circle) Gαolf. All cells were transfected with D1R and GloSensor-22F cAMP sensor. Values represent means ± SEM from three independent experiments each performed with four replicates. ***B***, Agonist-induced cAMP production of HEK293T/17 cells (filled circle) and *GNAS* KO cell line (open circle). All cells were transfected with D1R and GloSensor-22F cAMP sensor. Values represent means ± SEM from three independent experiments each performed with four replicates. ***C***, *GNAS* knockout cells were transfected with D1R and GloSensor-22F cAMP sensor together with (filled circle) or without (open circle) Gαolf. 100 μM dopamine-induced cAMP production was recorded. ***D***, Confirmation of selective D1R-Golf. D1R and Golf were transfected with GloSensor-22F cAMP sensor as indicated at the bottom of the bar graph. ***E***, Determination of EC50. Dose-response relationship was examined using *GNAS* KO cells transfected with D1R, Gαolf, and GloSensor-22F cAMP sensor.

Table S1. Genotype and clinical phenotype features of dystonia mutations used in the study.

Related to Figure 2.

Protein variant	Mutation site	Mutation type	Number of carrier reported	Ethnicity	Gender	Age of onset (years)	Dystonia distribution	Inheritance	Familial /sporadic	Reference
P102_ V104del	α-helical	In frame deletion	1	Caucasian	M	20	Fo	Het	Familial	Fuchs et al., 2013
F133L	α-helical	Missense	1	Brazilian	F	23	S	Het	Unknown	Dos Santos et al., 2016
V137M	α-helical	Missense	7	Caucasian	4xF and 3xM	7, 19, 22, 26, 31, 44, 50	6xS, 1xG	Het	Familial	Fuchs et al., 2013
V146M	α-helical	Missense	3	German	1xF	63	1xS	Het	Sporadic	Zech et al., 2014
E155K	α-helical	Missense	2	Caucasian	2xM	17, 18	1xFo, 1xS	Het	Familial	Fuchs et al., 2013
V172I	α-helical	Missense	1	Amish-Mennonites	F	21	S	Het	Sporadic	Saunders-Pullman et al., 2014
G213S	Switch II	Missense	1	German	M	40	Fo (no hyposmia)	Het	Unknown	Kumar et al., 2014
V228F	Switch II	Missense	5	African-American	3xF, 2xM	45, 50, 50, 63, N/A	1xFo, 3xS, 1xG with 2xMicrosmia	Het	Familial	Vemula et al., 2013
V234I	GTPase	Missense	1	Caucasian	M	36	Fo	Het	Sporadic	Putzel et al., 2016
S239N	Switch III	Missense	1	N/A	N/A	N/A	N/A	Het	Unknown	This study
A311T	GTPase	Missense	1	German	N/A	N/A	N/A	Het	Sporadic	Kumar et al., 2014
R329W	GTPase	Missense	2	Turkish	F	11 and 15	2xG	Ho	Familial	Masuho et al., 2016
A353T	TCAT motif	Missense	1	Japanese	F	44	Fo (no hyposmia)	Het	Unknown	Kumar et al., 2014
V354A	TCAT motif	Missense	1	Serbian	F	40	Fo	Het	Sporadic	Dobričić et al., 2014

M = Male, F = Female

Fo = Focal, S = Segmental, G = Generalized

Het = Heterozygous, Ho = Homozygous

Table S2: Criteria for deleteriousness classification of amino acid substitutions using different methods. Related to Figure 2.

Predictor	Classification criteria
Polyphen: Polyphen2 HDIV score (pp2_hdiv)	Deleterious: Probably damaging (pp2_hdiv ≥ 0.957), P: possibly damaging; Tolerated: Benign (pp2_hdiv ≤ 0.452) There were no DYT25 mutations in the possibly damaging category (0.453 ≤ pp2_hdiv ≤ 0.956) (Wang et al., 2010)
SIFT	Deleterious: SIFT score ≤ 0.05; Tolerated: SIFT score > 0.05 (Wang et al., 2010)
CADD	Deleterious: CADD PHRED-scaled score > 20; Tolerated: CADD PHRED-scaled score ≤ 20 (Kircher et al., 2014)
MetaLR	Deleterious: MetaLR score > 0.5; Tolerated: MetaLR score ≤ 0.5 (Dong et al., 2015)
REVEL	Deleterious: REVEL score > 0.5; Tolerated: REVEL score ≤ 0.5 (Ioannidis et al., 2016)
